# Designing a “better” brain: insights from experts and savants

**DOI:** 10.3389/fpsyg.2014.00470

**Published:** 2014-05-22

**Authors:** Fernand Gobet, Allan Snyder, Terry Bossomaier, Mike Harré

**Affiliations:** ^1^Department of Psychological Sciences, University of LiverpoolLiverpool, UK; ^2^Centre for the Mind, School of Medicine, University of SydneySydney, NSW, Australia; ^3^Centre for Research in Complex Systems, Faculty of Business, Charles Sturt UniversityBathurst, NSW, Australia; ^4^Complex Systems Research Group, Faculty of Engineering and IT, University of SydneySydney, NSW, Australia

**Keywords:** autism, brain, cognitive illusion, creativity, Einstellung effect, expertise, perception, savants

If we naively, simply scale up the brain—more of the same—we would not necessarily have a brain that is qualitatively better. Taking the analogy from visual ecology—the eagle—it could just see better at greater heights (Snyder and Miller, 1978)—more of the same!

Instead, let us ask how to design a brain that is qualitatively better in some sense, say at being creative. One bottleneck to creativity is our inability to see things in a new light, free of prior interpretations. Once a pattern is identified and labeled, it is difficult to identify different patterns and find alternative solutions. A classic example is the duck/rabbit illusion, where we can see either a duck or a rabbit but not be simultaneously aware of both. We are blinded by our mindsets, by our expertise! This presumably is a consequence of our hypothesis-driven, cognitive make up. Hypotheses are mental templates that encapsulate expectations of the world as derived from past experience—a brilliant strategy for coping rapidly with partial information in a dynamic but familiar environment (Snyder et al., 2004; Bossomaier et al., 2009). [Although we use the human brain as an example, there is considerably evidence that the concept-dominated architecture is characteristic of the mammalian brain, and possibly also in birds, in particular corvids (e.g., Emery and Clayton, 2004)].

Our brain can recall a seemingly unlimited number of meaningful patterns and labels, but often not the attributes that compose them. In contrast, some autistic savants can recall a seemingly unlimited amount of details, without any attempt to impose meaning (e.g., Wiltshire, 1989). But, savants are less prone to cognitive illusions (Bogdashina, 2003) and that fact gives us a clue for a “better” brain—a brain that is hypothesis driven, but is resilient to cognitive illusions, a brain that can in addition see the world with direct perception and thus open to alternative interpretations (Snyder, 2009).

A better brain—natural or artificial—would have tremendous implications for our world. Most importantly, it would boost creativity, both in art and in science. Better decisions would be made in politics and business, and long-standing issues such as pollution and hunger would be more likely to be solved. How do we go about to developing such a brain?

A possible strategy is to start from the things humans are doing so well as a result of our adaptation to the environment due to evolutionary pressure, and then to consider why the products of this adaptation sometimes are associated with penalties. Finding a way to fix these instances of penalties in humans leads to insights that can be further applied to the design of better brains.

As a first example, consider visual perception. Overall, our eye and our visual cortex, honed by millions of years of evolution, work very well. However, human visual perception can be fooled surprisingly easily by perceptual and cognitive illusions. We can see things that do not exist (e.g., Escher's impossible figures), do not see things that exist (e.g., reading words rather than seeing black and white pixels), see two objects from the same drawing (e.g., the duck-rabbit illusion), and grossly misjudge the dimensions of objects (e.g., the Ebbinghaus and Ponzo illusions). How can we avoid these illusions? What would this tell us about brain design?

As a second example, consider expertise. Experts can show extreme adaptations to their environment and are capable of great achievements (Ericsson et al., 2006; Didierjean and Gobet, 2008). Tennis players can return services with balls hit at more than 200 km/h, and they can even counter-attack when doing so. Mnemonists can memorize more than 100 digits read at 1 s each. Some chess players can play more than 30 games simultaneously, without seeing the board and the pieces. But expertise sometimes fails. In their study on the Einstellung effect in chess, Bilalić et al. (2008a,b, 2010) showed that even experts can fail to find an optimal solution when a common solution comes first to their mind. The effect is surprisingly powerful: compared to control positions, players lose about 1 standard deviation in skill when showing the Einstellung effect. This illustrates the strength of the schemas we hold in long-term memory and the power that our preconceptions have on our mind.

What are the solutions to perceptual/cognitive illusions, such as those displayed in visual illusions and in the Einstellung effect? Intuitively, there are two main approaches. The first one is to use more knowledge (i.e., scaling up) and could be called “quantitative scaling.” It could be summarized by the phrase “more of the same.” This is the standard approach in computer science and artificial intelligence.

Quantitative scaling has had some tremendous successes in artificial intelligence, such as the victory of Deep Blue against chess world champion Kasparov in 1997 (Campbell et al., 2002). But increase of knowledge does not always provide a solution; for example, many visual illusions persist in spite of our knowledge that they exist. Indeed, quantitative scaling has also met with problems in artificial intelligence. Most notably, it has failed in many attempts, such as playing Go at master level (Gobet et al., 2004; Hsu, 2007), providing computers with common sense (McCarthy, 2007; Sarbo, 2007), and in general developing genuine, domain-general intelligence and creativity (Bridewell and Langley, 2010; Jennings, 2010).

A second approach could be called “qualitative scaling.” In essence, this leads to the definition and use of new conceptual spaces. To get to these new spaces, we need to break apart the existing conceptual structures, whereas in quantitative scaling we build increasingly elaborate concepts on top of what we have already. A classic example is Einstein's conjecture that the speed of light was constant in all inertial reference frames, leading to conclusions very different to those of Newtonian physics. Serialism, cubism or simply Dadaism itself illustrate this notion in the arts.

We know that individuals with autism are less susceptible to some illusions (Walter et al., 2009; Mitchell et al., 2010), and that they process information in a less holistic way (Nakahachi et al., 2008), for example paying more attention to details, which can result in savant skills such as extreme memory for detail and speed in counting objects (numerosity) (Soulieres et al., 2010). We also know that some savant skills can be artificially induced in normal individuals, for example by low-frequency repetitive transcranial magnetic stimulation (Snyder et al., 2006; Boggio et al., 2009; Snyder, 2009) or transcranial direct current stimulation (Chi and Snyder, 2012).

Our key argument is that our normal cognition, while very efficient, tends to develop cognitive mind sets. Breaking these mind sets can help explore new conceptual spaces, and thus be more creative. Rather than reorganizing knowledge in some superficial way, we propose two radical approaches. The first is to inhibit some concepts or a class of concepts (e.g., a group of concepts that are strongly connected or the most likely concepts in a given situation). The second is to avoid concepts altogether (or at least as much as possible), by using raw perception instead, thus imitating the autistic mind. Our proposal is thus that creativity can be boosted by decreasing conceptual processing and increasing the role of low-level perceptual processing.

Our proposal raises intriguing issues. What is the link between concepts and raw perception? Are they two discrete states, or are they part of a more graded space? How can “raw perception” mitigate or even eliminate Einstellung-like effects? Is raw perception enough? Autism seems to offer a counter-example, where the lack of use of concepts leads to serious intellectual and social impairments. But is it necessarily so?

The history of human thought provides us with creativity examples where the inhibiting-concept strategy was used (possibly unconsciously) and worked. When 2005 Nobel Laureates Marshall and Warren (1984) correctly proposed that stomach ulcers were caused by helicobacter pylori rather than by excess acid, they had to jettison a whole raft of concepts. There are also examples of this in AI research—for instance computer chess and checkers, where new profound insights were gained from brute force search, without using sophisticated concepts. In the latter case, the literal perception used by computers, which is often derided, turns out to be a strength. The cost of deleting concepts might also be studied. If the concepts that are inhibited are infrequent and are not the building blocks of a large number of other concepts, the benefits might outweigh the costs. But what is the threshold? Another solution with artificial systems is parallelism. A possibility would be that the original conceptual base stays online while concepts are inhibited in a copy of the original base that operates offline.

The examples in this article focus on finding novel creative solutions without being bound by prior knowledge. It is an open question as to exactly what a better brain would be: more creative, more efficient, more rational, more adaptive, more altruistic? Answering this question is a huge challenge in itself, and we should be alert to our own mindset in defining the space of possible answers.

The implications for the study of human cognition and the psychology expertise in particular are profound. A better brain would shed considerable but also cruel light on the limits of human cognition and expertise. We already had a preview of this with developments in computer chess. No players, even world champion Magnus Carlsen, can compete with computers nowadays. Computers sometimes find moves that are considered by humans as highly creative, although some of these moves are just beyond human discovery. In addition, computers have led to re-evaluations of large aspects of the game, in particular openings and endgames, which humans had researched for centuries. Be ready to be surprised with better brains!

Irrespective of possible benefits for science and technology, including artificial intelligence, our proposal for a better brain raises important questions for the nature of the human mind. Are qualitative scaling and quantitative scaling really in opposition? Is it adaptive to inhibit some concepts “just” to be creative? Why has such a system not evolved? Is it because, now, we have the luxury to be creative—courtesy of cultural evolution?

## Conflict of interest statement

The authors declare that the research was conducted in the absence of any commercial or financial relationships that could be construed as a potential conflict of interest.
